# Supplementary material for: Investigation of the Variability of Alkaloids in Buxus sempervirens L. Using Multivariate Data Analysis of LC/MS Profiles
Source: Molecules. 2021 Dec 23;27(1):82. doi: 10.3390/molecules27010082 (PMC8746436; doi:10.3390/molecules27010082)
Supplement: Supplementary file 1 [file molecules-27-00082-s001.zip › molecules-1513766-supplementary.pdf]

molecules

Supplementary Materials

Investigation of the Variability of Alkaloids in *Buxus sempervirens* L. Using Multivariate Data Analysis of LC/MS Profiles

Lara U. Szabó [1] and Thomas J. Schmidt [1,*]

[1] Institute of Pharmaceutical Biology and Phytochemistry (IPBP), PharmaCampus, University of Münster, PharmaCampus Corrensstraße 48, D-48149 Münster, Germany; lszabo@uni-muenster.de
* Correspondence: thomschm@uni-muenster.de; Tel.: +49-251-83-33378

Citation: Szabó, L.-U.; Schmidt, T. J. Investigation of the Variability of Alkaloids in *Buxus sempervirens* L. Using Multivariate Data Analysis of LC/MS Profiles. *Molecules* **2022**, *27*, 82. https://doi.org/10.3390/molecules27010082

Academic Editor: Vera Muccilli, Monica Scognamiglio and Julien Wist

Received: 1 December 2021
Accepted: 21 December 2021
Published: 23 December 2021

Publisher's Note: MDPI stays neutral with regard to jurisdictional claims in published maps and institutional affiliations.

Copyright: © 2021 by the authors. Licensee MDPI, Basel, Switzerland. This article is an open access article distributed under the terms and conditions of the Creative Commons Attribution (CC BY) license (https://creativecommons.org/licenses/by/4.0/).

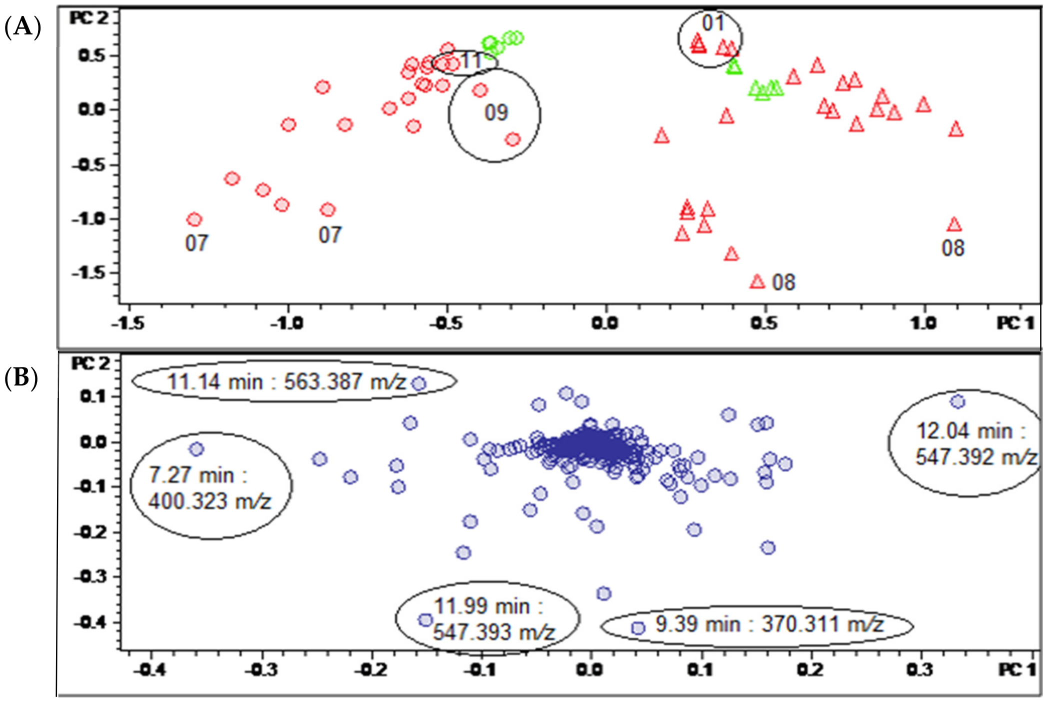

Figure S1. (**A**) Scores and (**B**) loadings plot, no scaling (*none*), PC1 (28.0%) and PC2 (22.6%). Legend: red = leaf; green = twig; circle = *B. sempervirens var. suffruticosa* L.; triangle = *B. sempervirens var. arborescens* L.; number = month; circle = related technical replicates (n = 2; QC n = 4).

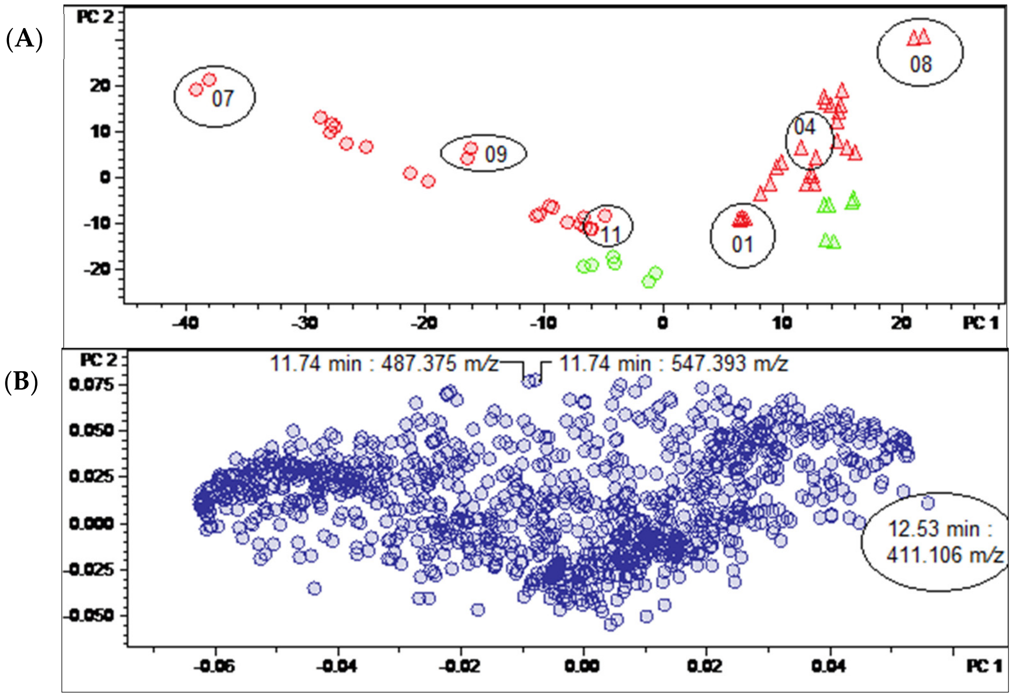

Figure S2. (**A**) Scores and (**B**) loadings plot, scaling: *unit variance*, PC1 (20.4%) and PC2 (12.6%). Legend: red = leaf; green = twig; circle = *B. sempervirens var. suffruticosa* L.; triangle = *B. sempervirens var. arborescens* L.; number = month; circle = related technical replicates (n = 2; QC n = 4).

(**A**)

(**B**)

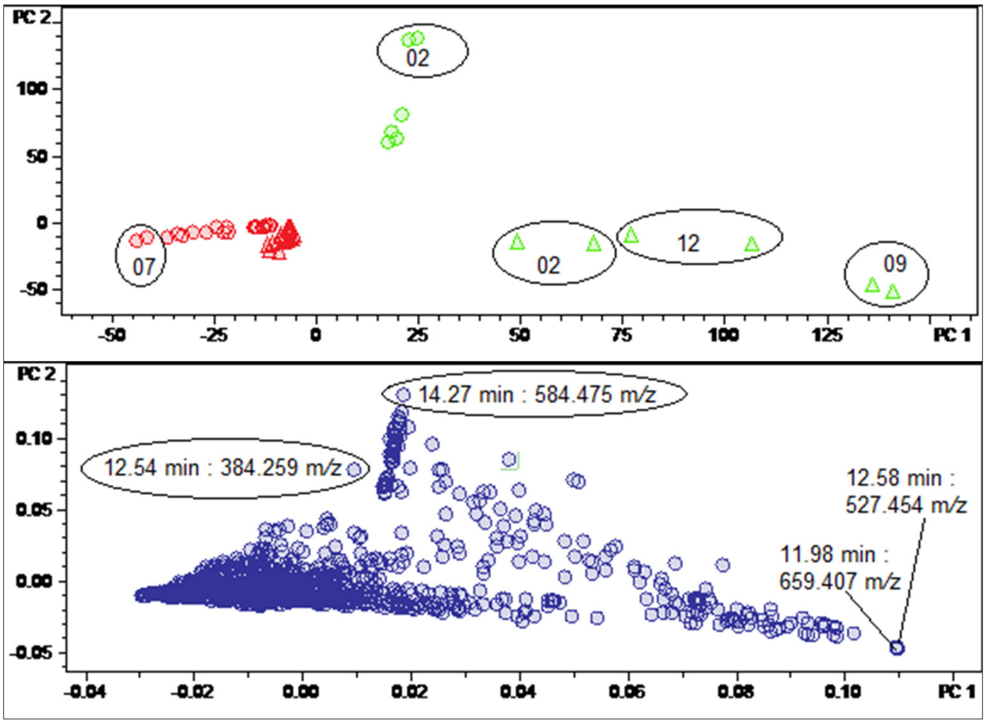

Figure S3. (**A**) Scores and (**B**) loadings plot, scaling: *level*, PC1 (13.8%) and PC2 (11.5%). Legend: red = leaf; green = twig; circle = *B. sempervirens var. suffruticosa* L.; triangle = *B. sempervirens var. arborescens* L.; number = month; circle = related technical replicates (n = 2; QC n = 4).

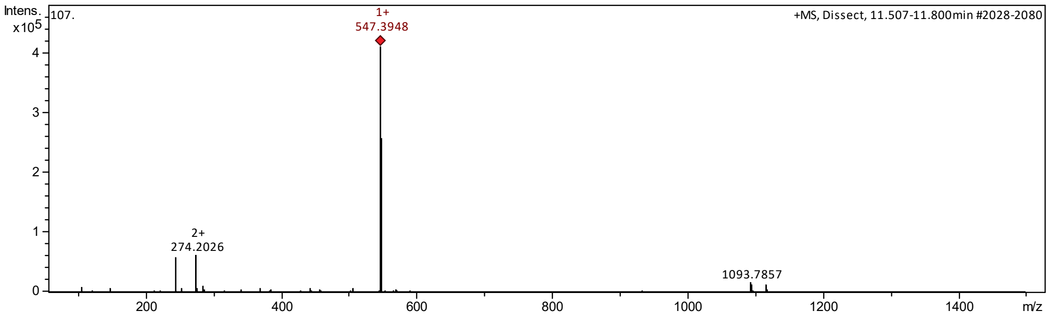

Figure S4. +ESI-QqTOF MS spectrum of compound **22** (N-benzoyl-O-acetylbuxodienine-E [3]); m/z 274.2026 $[M+2H]^{2+}$, 547.3948 $[M+H]^+$ and 1093.7857 $[2M+H]^+$.

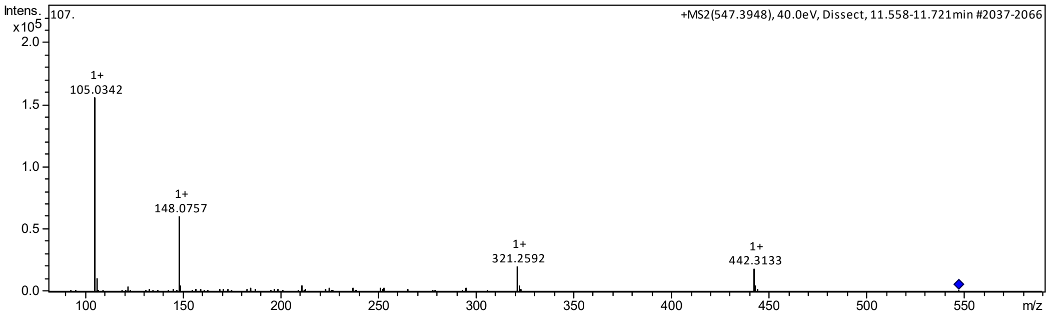

Figure S5. +ESI-QqTOF MS/MS spectrum of compound **22** (N-benzoyl-O-acetylbuxodienine-E [3]). The fragmentation pathway was already reported in [3].

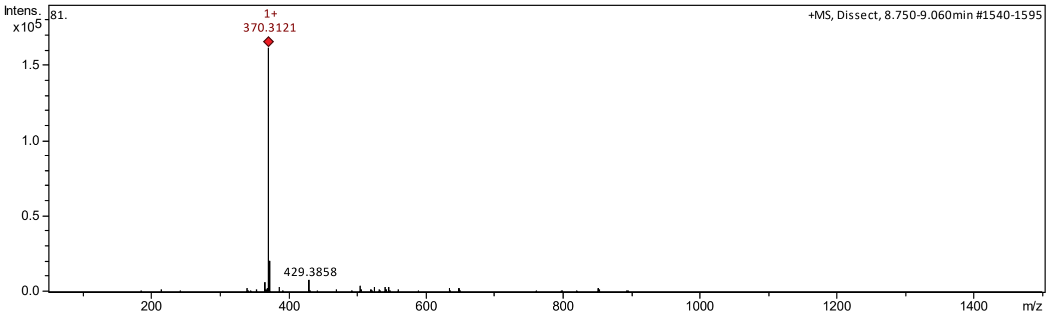

Figure S6. +ESI-QqTOF MS spectrum of compound **13** ((*E*)-Cyclobuxophyllinine-M [3]); m/z 370.3121 $[M+H]^+$.

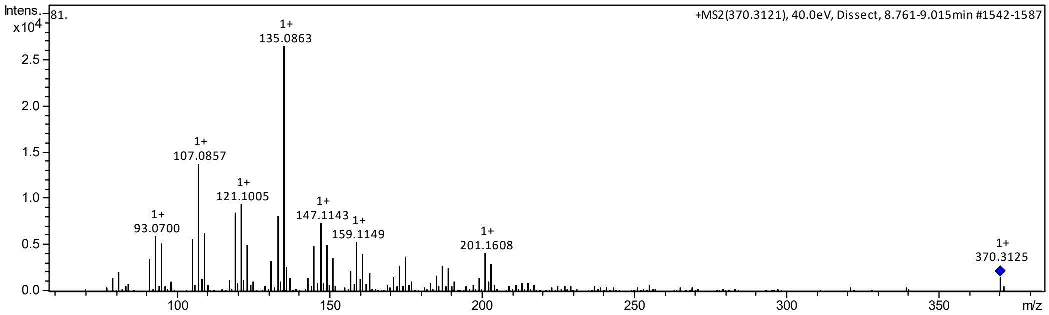

Figure S7. +ESI-QqTOF MS/MS spectrum of compound **13** ((*E*)-Cyclobuxophyllinine-M [3]).

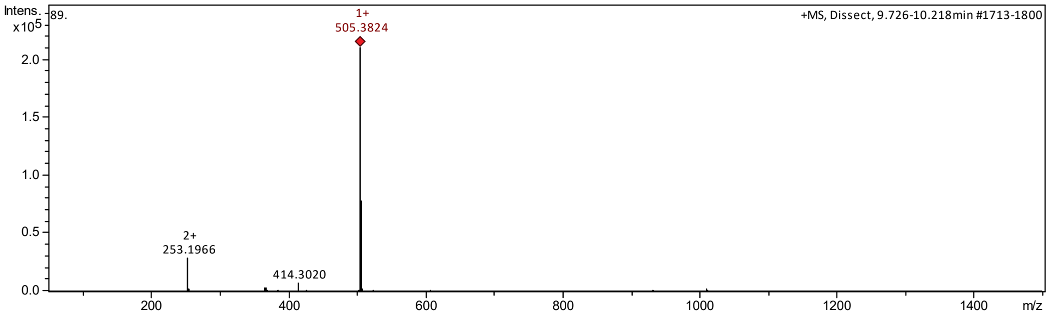

Figure S8. +ESI-QqTOF MS spectrum of compound **26** (N-benzoyl-cycloxo-buxine-F [17]); m/z 253.1966 $[M+2H]^{2+}$ and 505.3824 $[M+H]^{+}$.

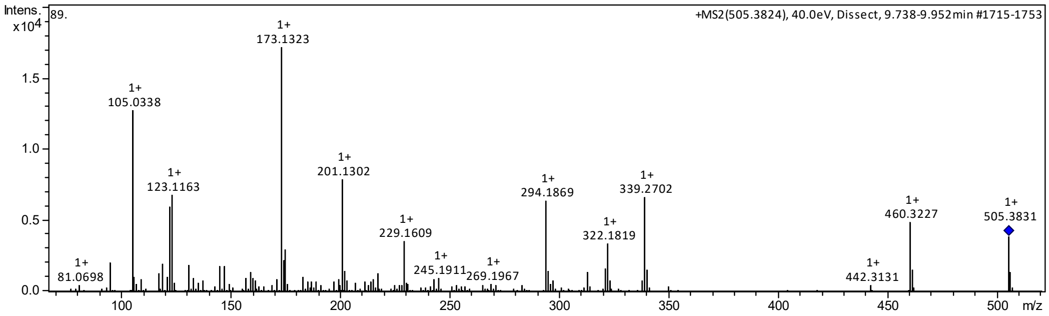

Figure S9. +ESI-QqTOF MS/MS spectrum of compound **26** (N-benzoyl-cycloxo-buxine-F [17]).

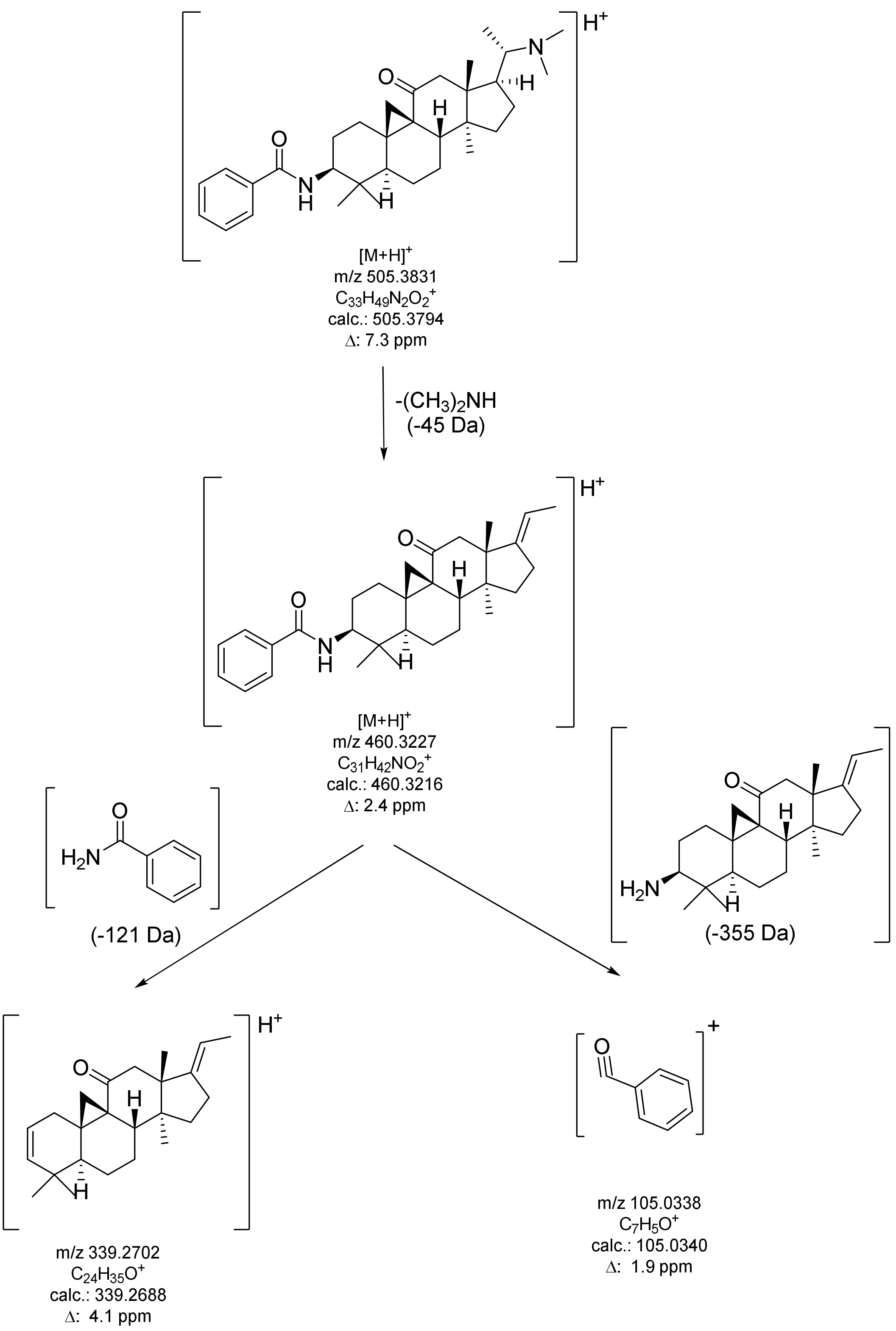

Figure S10. Possible fragmentation pathway of the [M + H]+ ion of compound **26** (N-benzoyl-cycloxo-buxine-F).

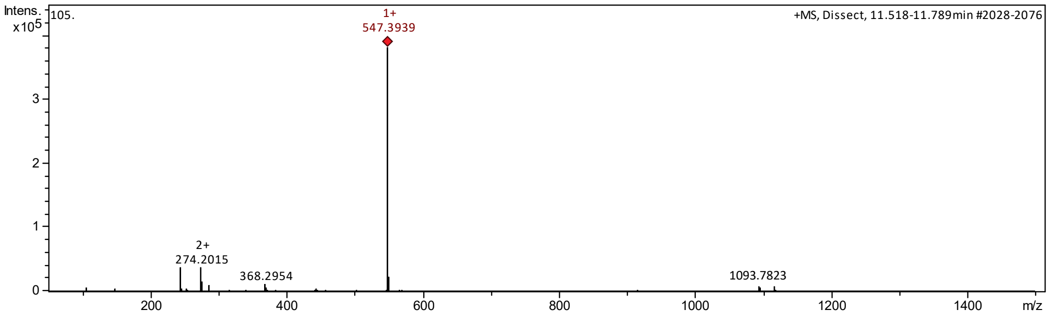

Figure S11. +ESI-QqTOF MS spectrum of compound **23** (N-benzoyl-O-acetylbuxadine-E [3]); m/z 274.2015 [M+2H]$^{2+}$, 547.3939 [M+H]$^{+}$ and 1093.7823 [2M+H]$^{+}$.

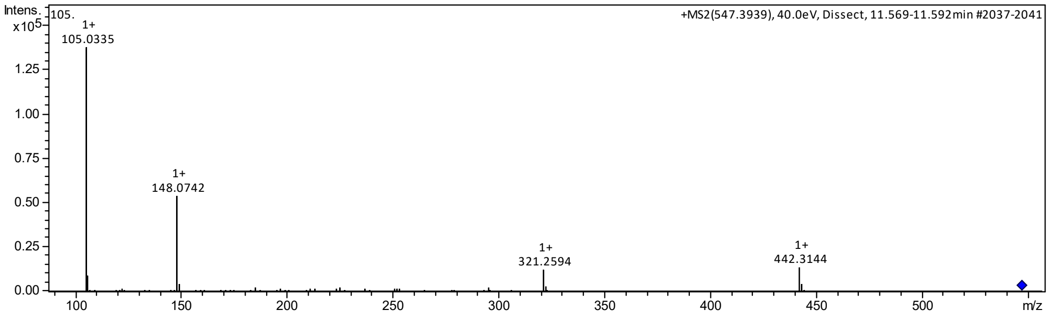

Figure S12. +ESI-QqTOF MS/MS spectrum of compound **23** (N-benzoyl-O-acetylbuxadine-E [3]). The fragmentation pathway was already reported in [3].

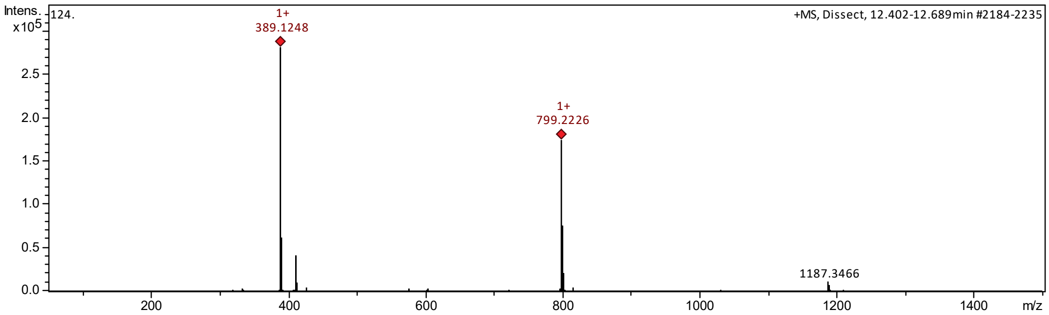

Figure S13. +ESI-QqTOF MS spectrum of compound **27**; m/z 389.1248 [M+H]$^{+}$.

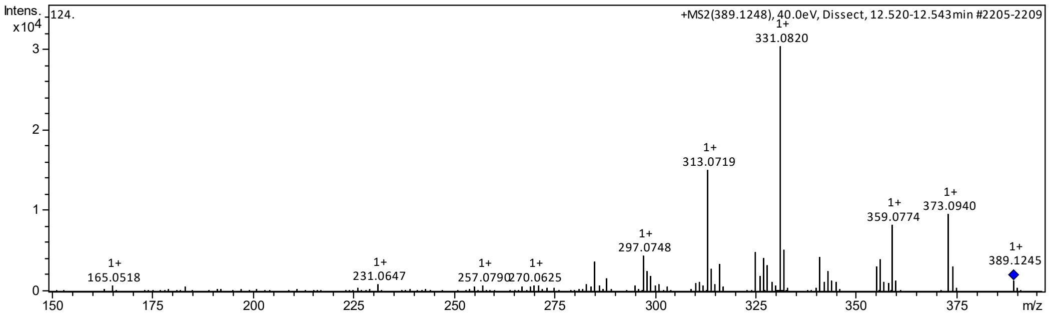

Figure S14. +ESI-QqTOF MS/MS spectrum of compound **27**.

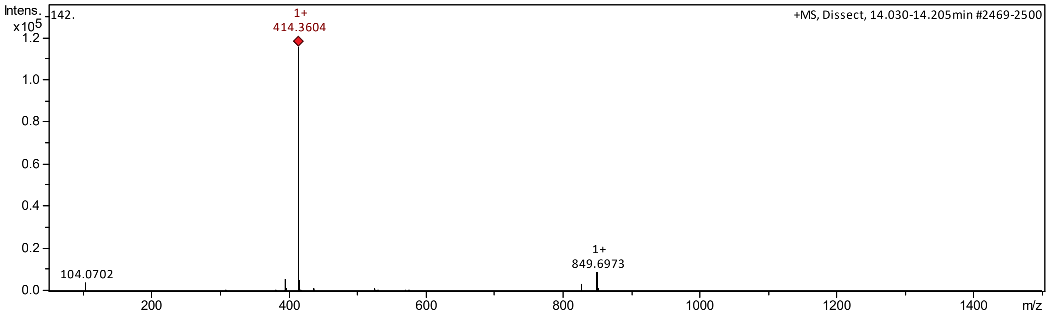

Figure S15. +ESI-QqTOF MS spectrum of compound **28**; m/z 414.3604 [M+H]$^+$.

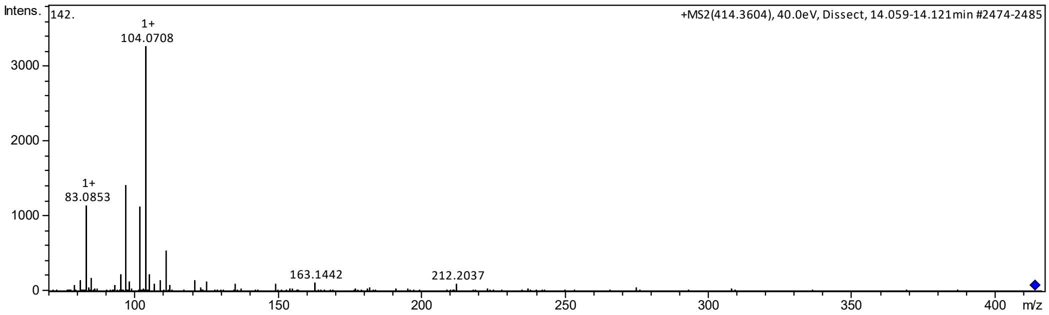

Figure S16. +ESI-QqTOF MS/MS spectrum of compound **28**.

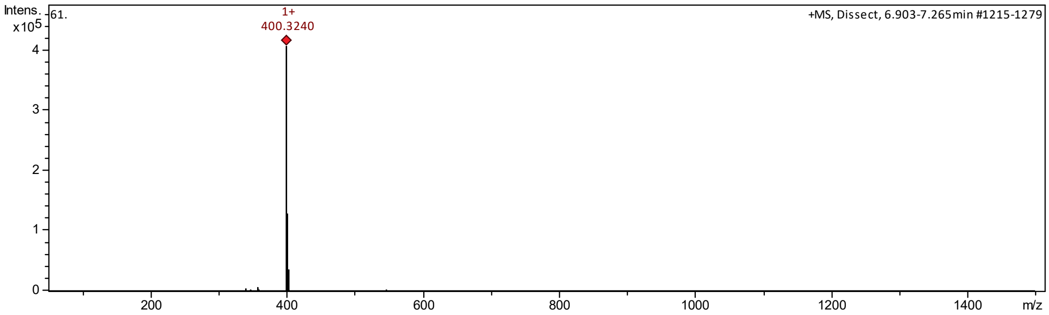

Figure S17. +ESI-QqTOF MS spectrum of compound **12** (N_b-dimethylcycloxobuxoviricine [3]); m/z 400.3240 [M+H]$^+$.

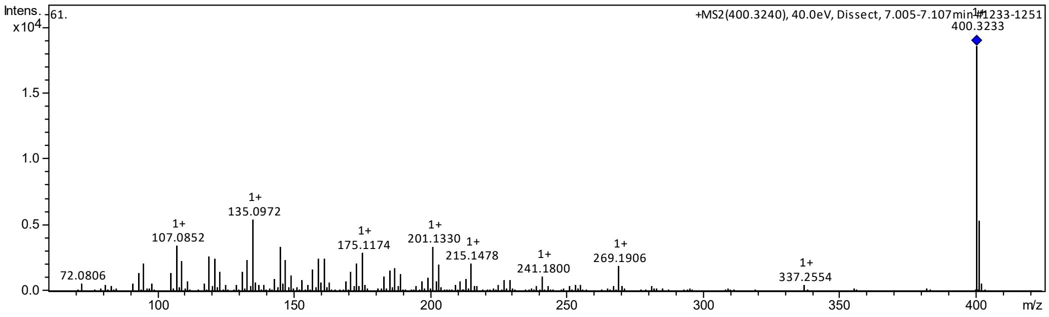

Figure S18. +ESI-QqTOF MS/MS spectrum of compound **12** (N_b-dimethylcycloxobuxoviricine [3]).

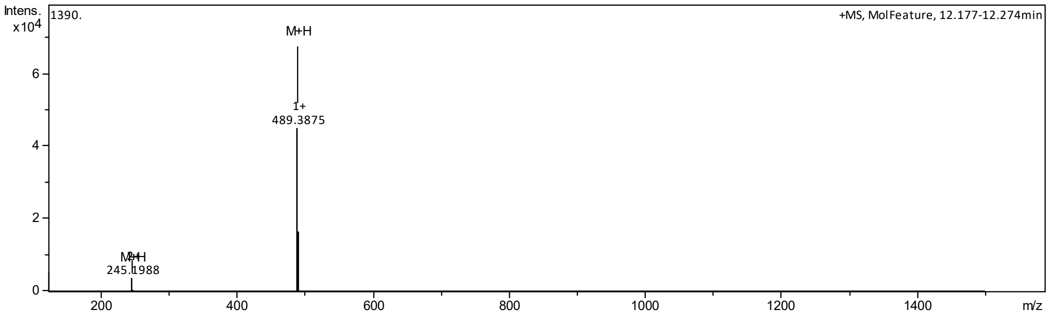

Figure S19. +ESI-QqTOF MS spectrum of compound **29**; m/z 245.1988 $[M+2H]^{2+}$ and 489.3875 $[M+H]^{+}$.

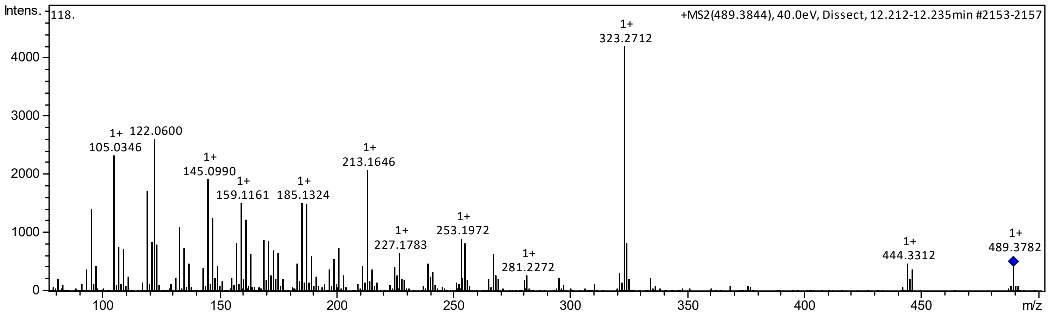

Figure S20. +ESI-QqTOF MS/MS spectrum of compound **29**.

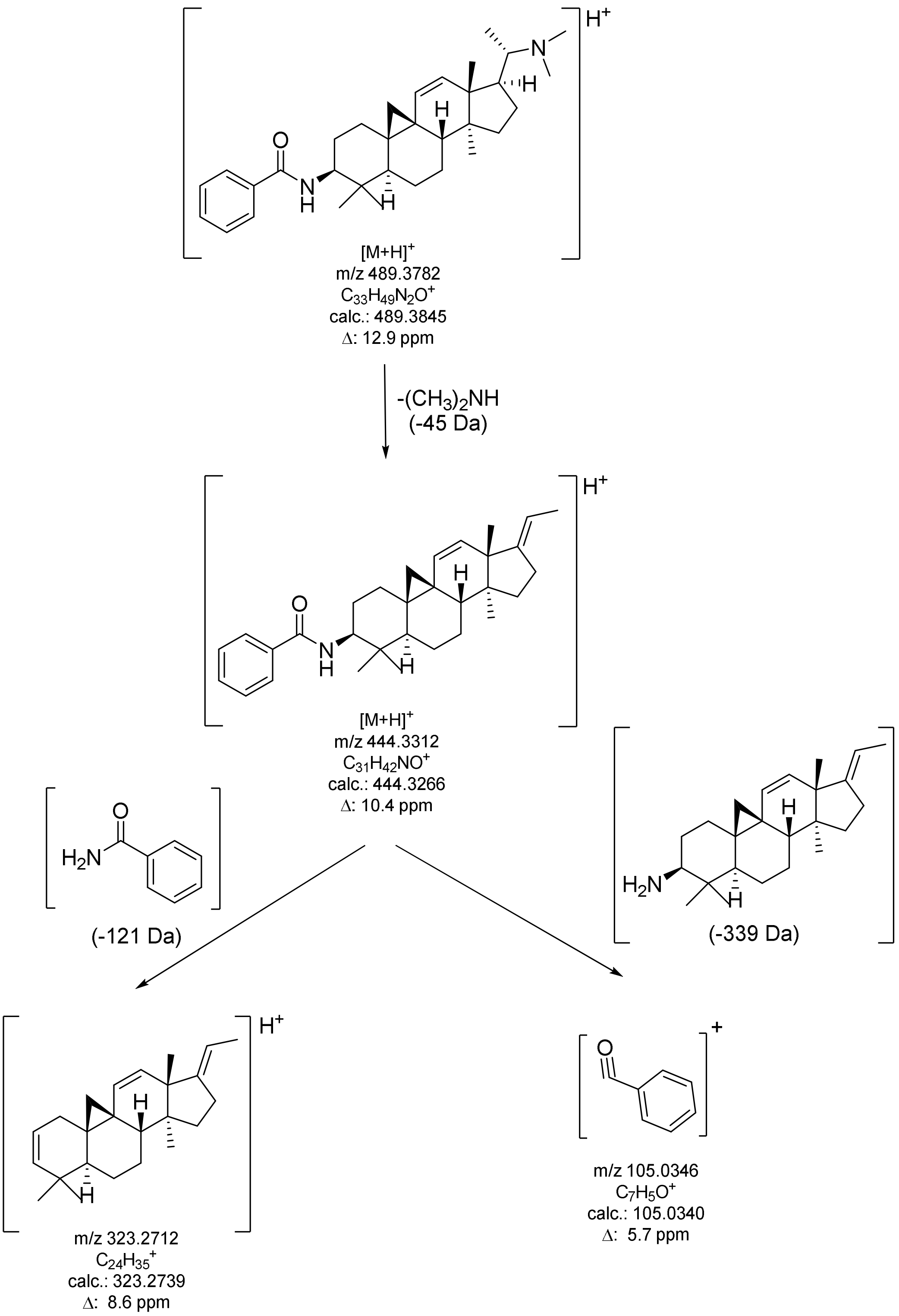

Figure S21. Possible fragmentation pathway of the [M + H]+ ion of compound **29**. (The postulated position of the substituents and double bond is based on the already known structures of *Buxus*-alkaloids e.g. compound **26**).

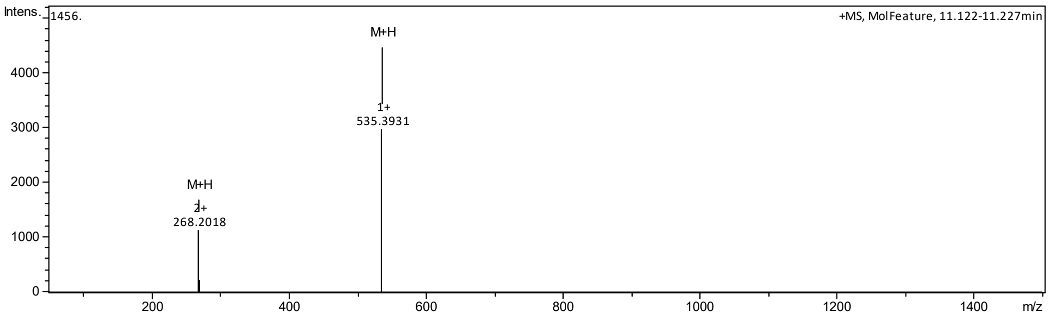

Figure S22. +ESI-QqTOF MS spectrum of compound **30** (Buxruguloid-B [19]); m/z 268.2018 $[M+2H]^{2+}$ and 535.3931 $[M+H]^{+}$.

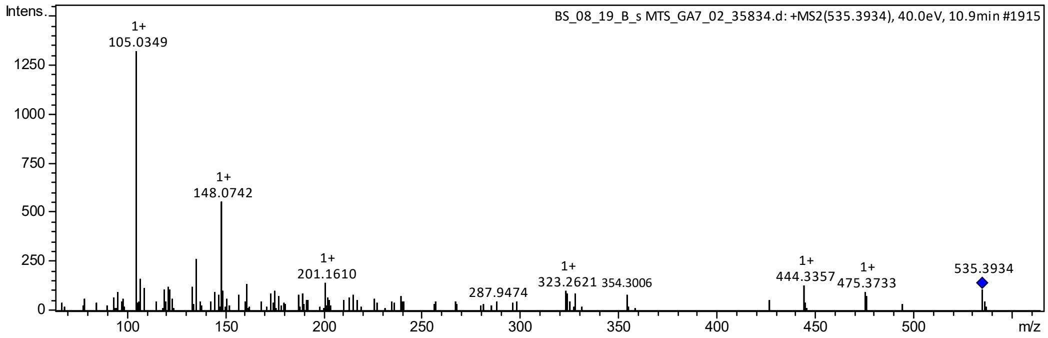

Figure S23. +ESI-QqTOF MS/MS spectrum of compound **30** (Buxruguloid-B [19]).

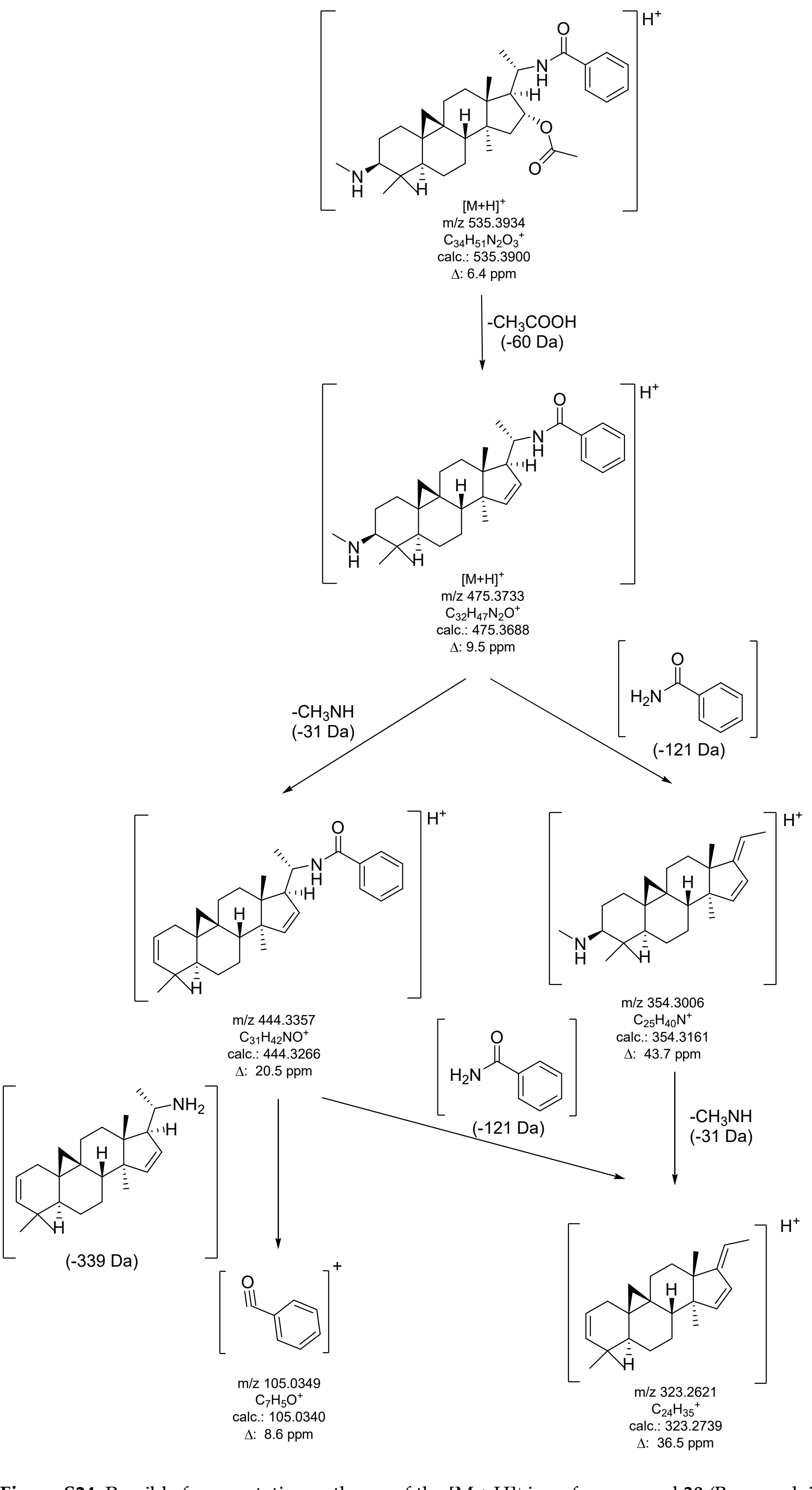

Figure S24. Possible fragmentation pathway of the [M + H]+ ion of compound **30** (Buxruguloid-B).

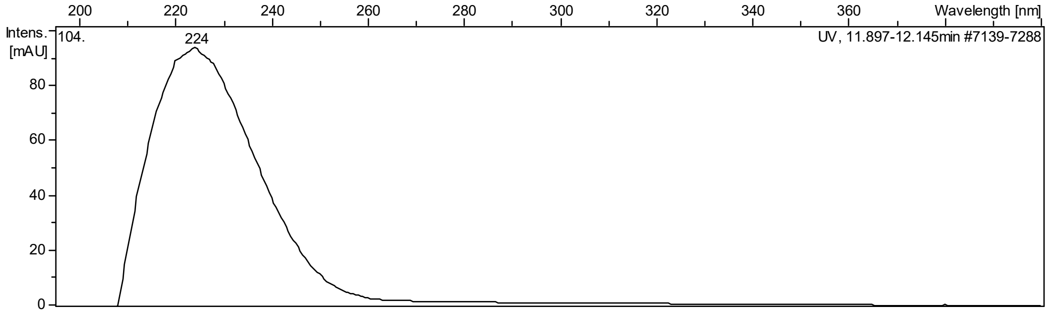

Figure S25. UV spectrum of compound **31**.

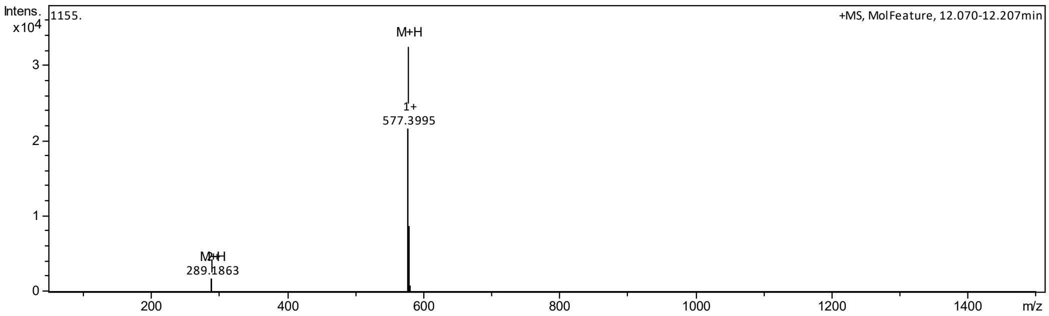

Figure S26. +ESI-QqTOF MS spectrum of compound **31**; m/z 289.1863 $[M+2H]^{2+}$ and 577.3995 $[M+H]^{+}$.

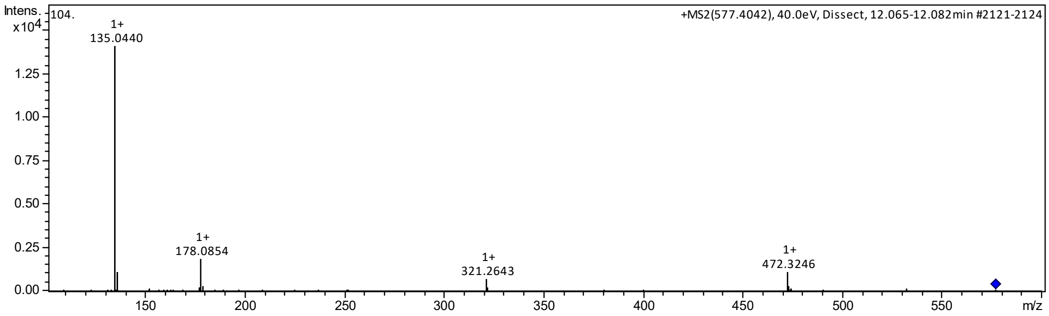

Figure S27. +ESI-QqTOF MS/MS spectrum of compound **31**.

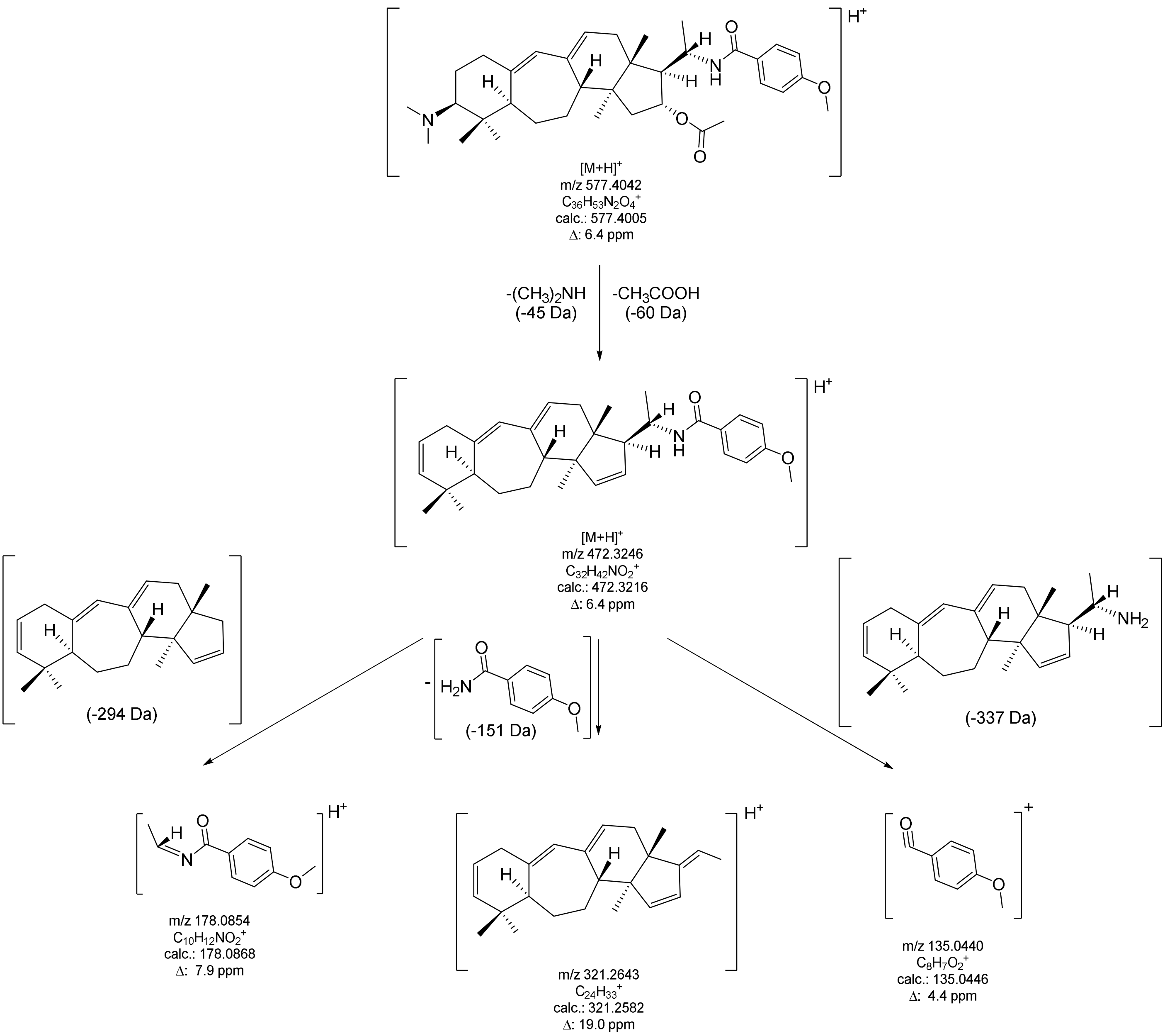

Figure S28. Possible fragmentation pathway of the [M + H]+ ion of compound **31**. (The postulated position of the ester group is based on the already known structures of compound **22** and **23**).

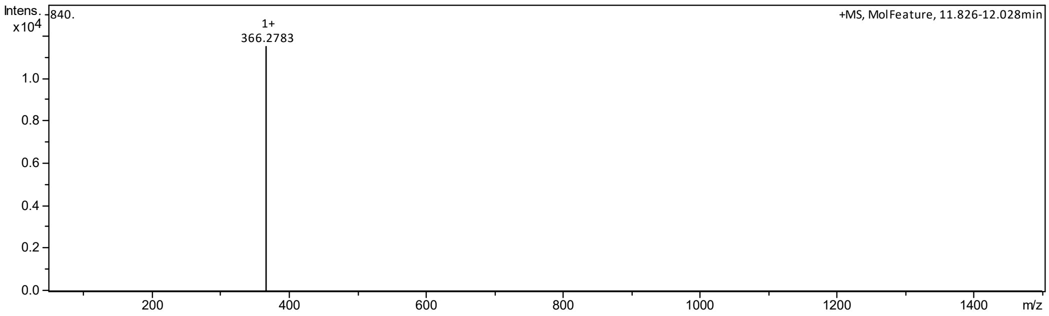

Figure S29. +ESI-QqTOF MS spectrum of compound **32** (Spirofornabuxin [21]); m/z 366.2783 [M+H]+.

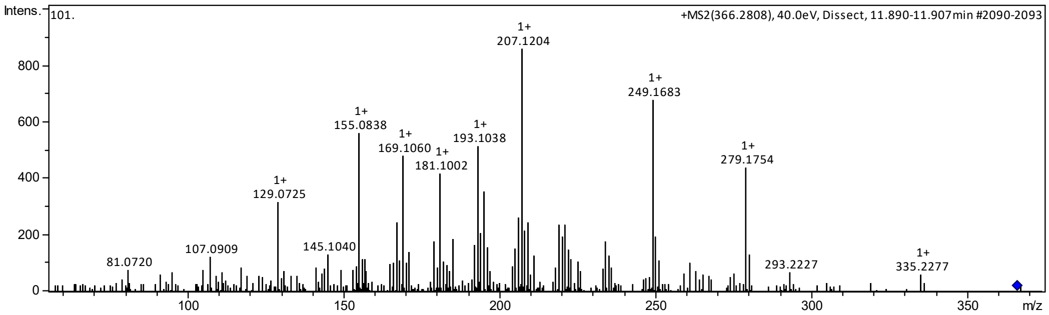

Figure S30. +ESI-QqTOF MS/MS spectrum of compound **32** (Spirofornabuxin [21]).

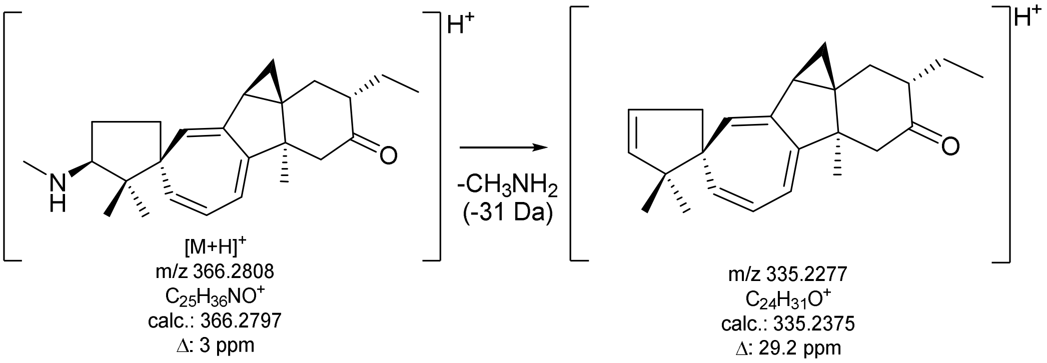

Figure S31. Possible beginning of the fragmentation pathway of the [M + H]+ ion of compound **32** (Spirofornabuxin).

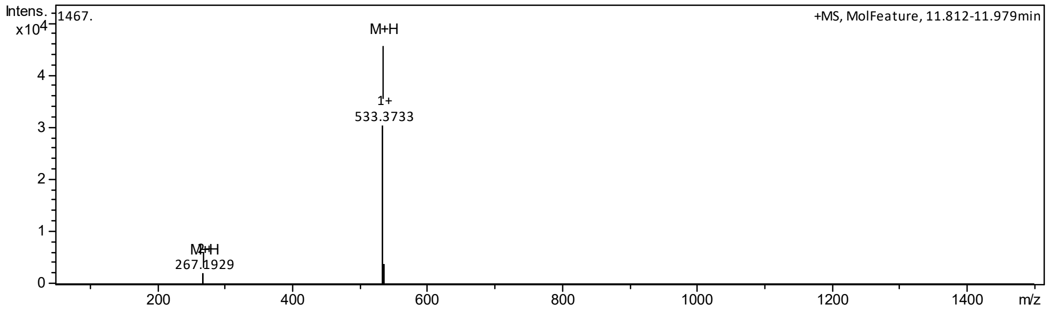

Figure S32. +ESI-QqTOF MS spectrum of compound **33** (Buxusemine-H [22]); m/z 267.1929 $[M+2H]^{2+}$ and 533.3733 $[M+H]^{+}$.

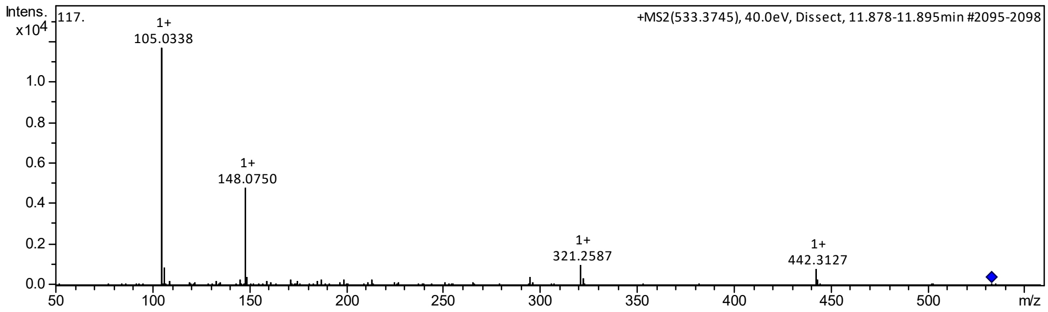

Figure S33. +ESI-QqTOF MS/MS spectrum of compound **33** (Buxusemine-H [22]).

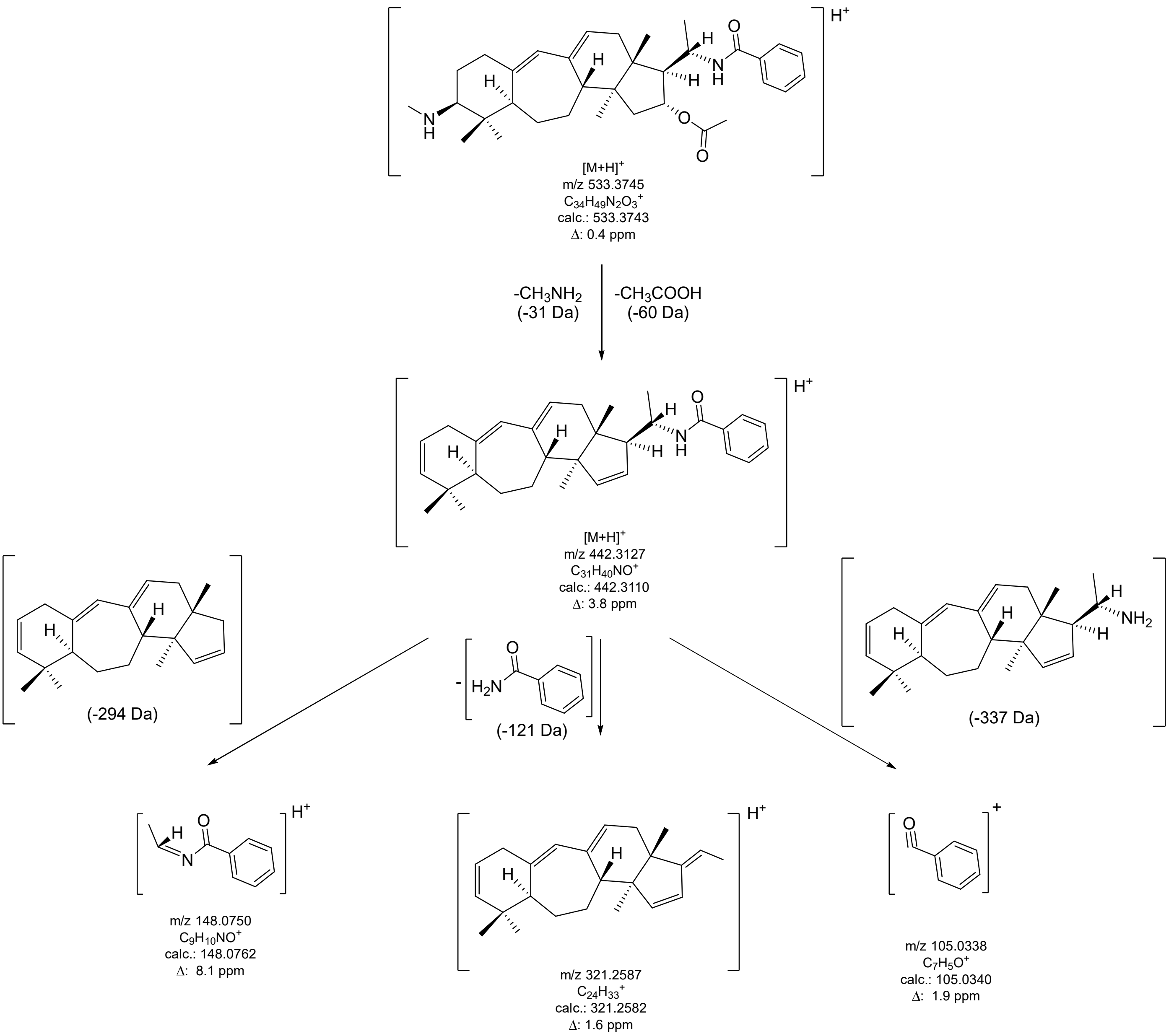

Figure S34. Possible fragmentation pathway of the [M + H]+ ion of compound **33** (Buxusemine-H).

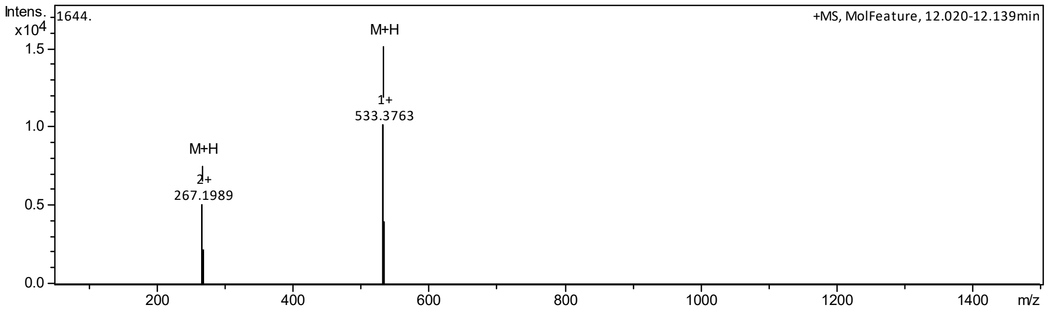

Figure S35. +ESI-QqTOF MS spectrum of compound **34** (Buxusemine-L [22]); m/z 267.1989 $[M+2H]^{2+}$ and 533.3763 $[M+H]^{+}$.

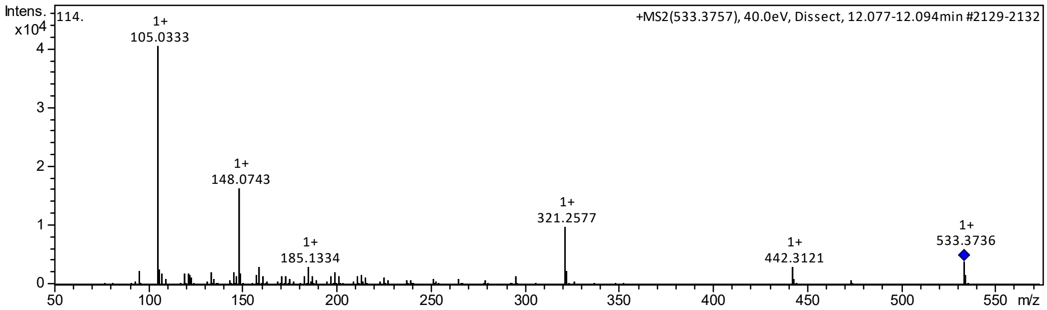

Figure S36. +ESI-QqTOF MS/MS spectrum of compound **34** (Buxusemine-L [22]).

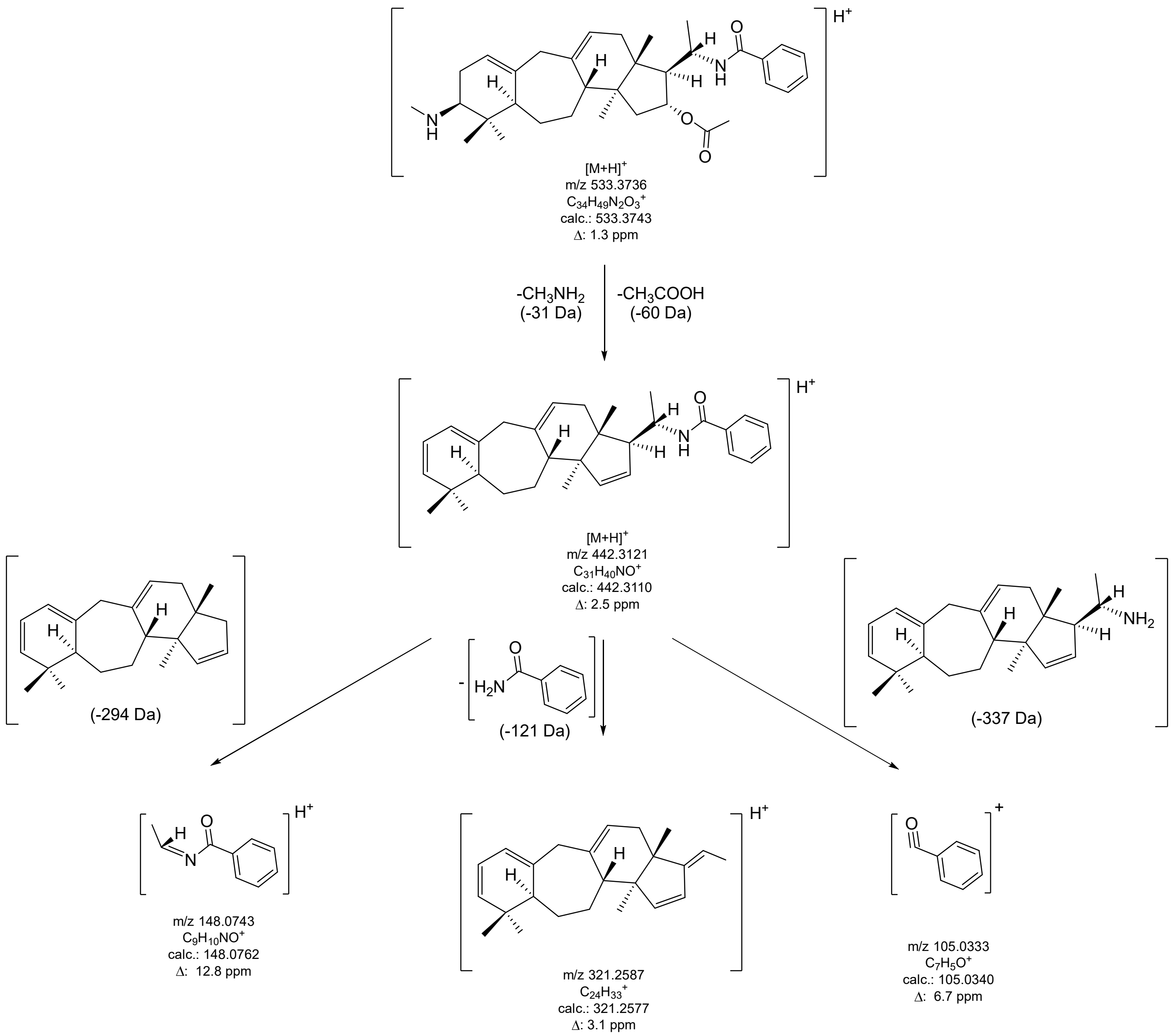

Figure S37. Possible fragmentation pathway of the [M + H]+ ion of compound **34** (Buxusemine-L).

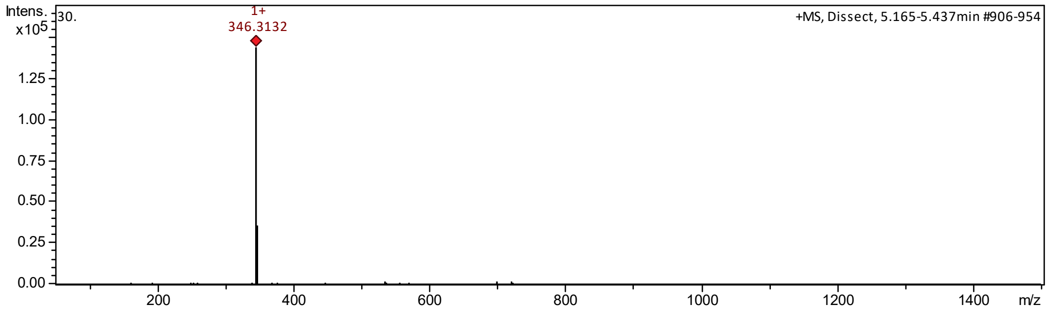

Figure S38. +ESI-QqTOF MS spectrum of compound **19** (Irehine [3, 23]); m/z 346.3132 [M+H]$^+$.

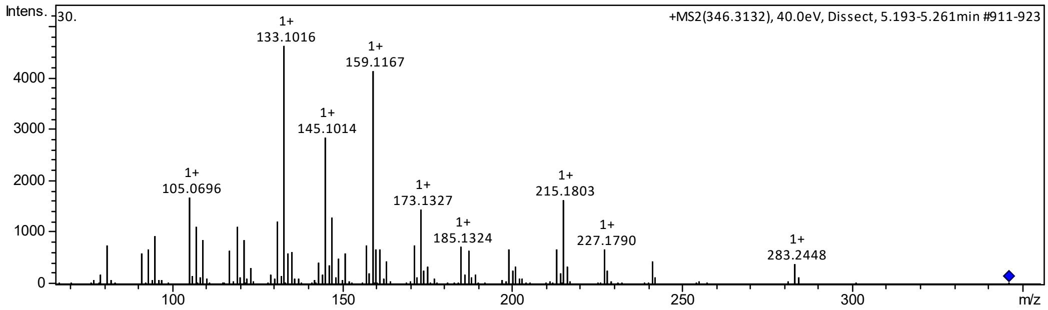

Figure S39. +ESI-QqTOF MS/MS spectrum of compound **19** (Irehine [3, 23]).

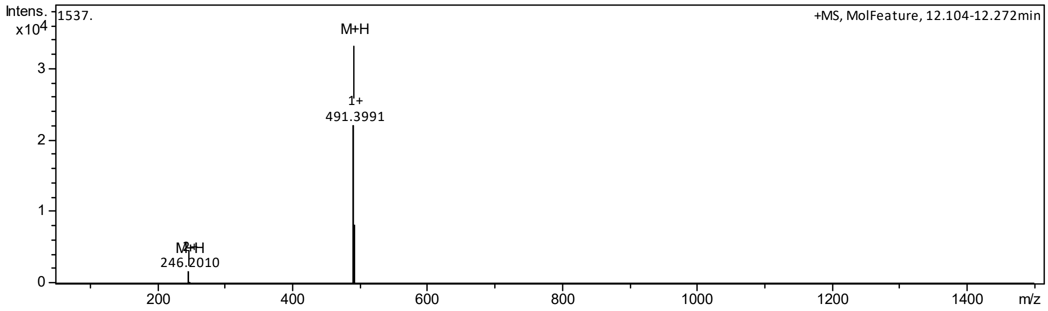

Figure S40. +ESI-QqTOF MS spectrum of compound **35**; m/z 246.2010 [M+2H]$^{2+}$ and 491.3991 [M+H]$^+$.

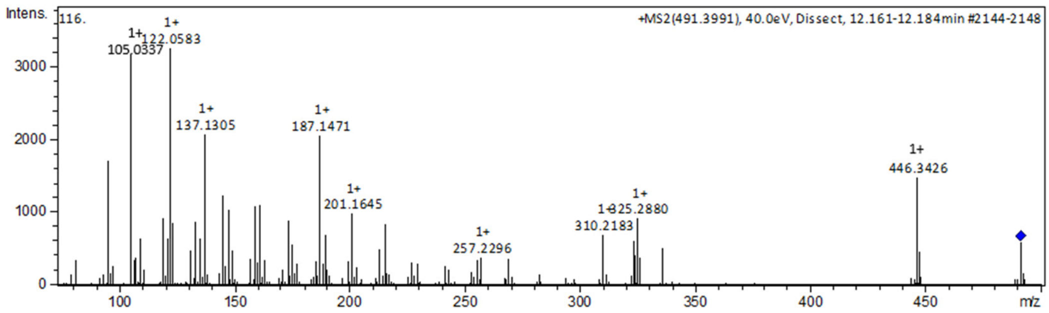

Figure S41. +ESI-QqTOF MS/MS spectrum of compound **35**.

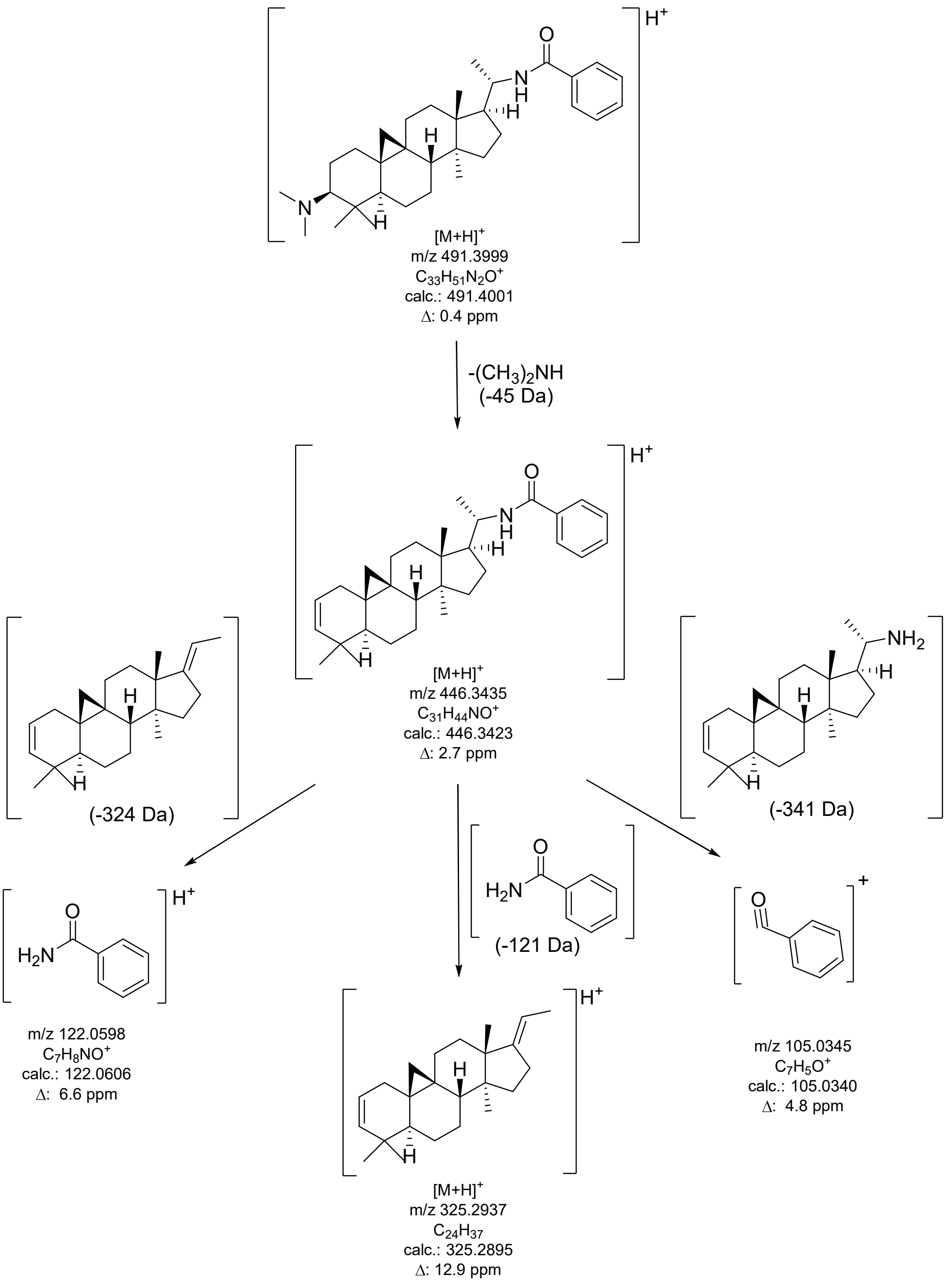

Figure S42. Possible fragmentation pathway of the [M + H]+ ion of compound **35**. (The postulated position of the amide group is based on the already known structures of compound **30**).

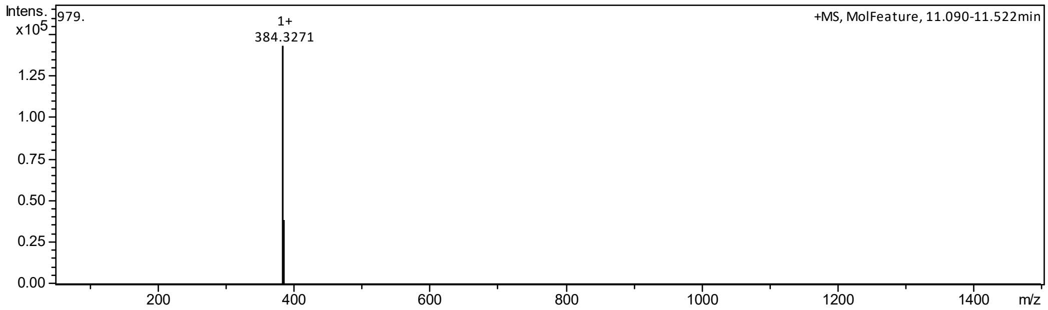

Figure S43. +ESI-QqTOF MS spectrum of compound **36**; m/z 384.3271 [M+H]⁺.

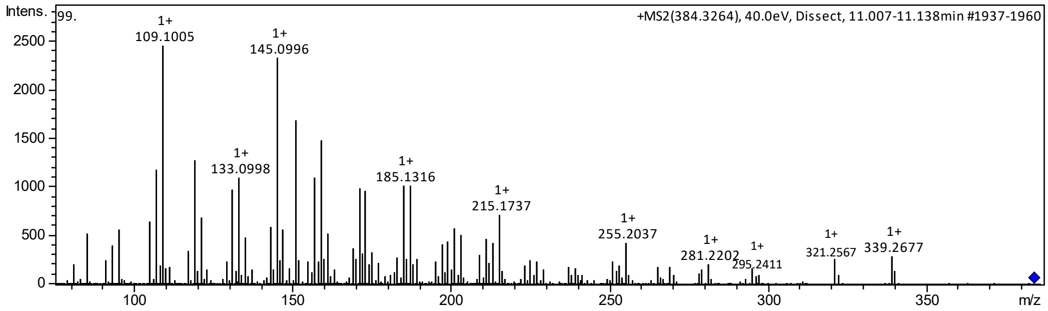

Figure S44. +ESI-QqTOF MS/MS spectrum of compound **36**.

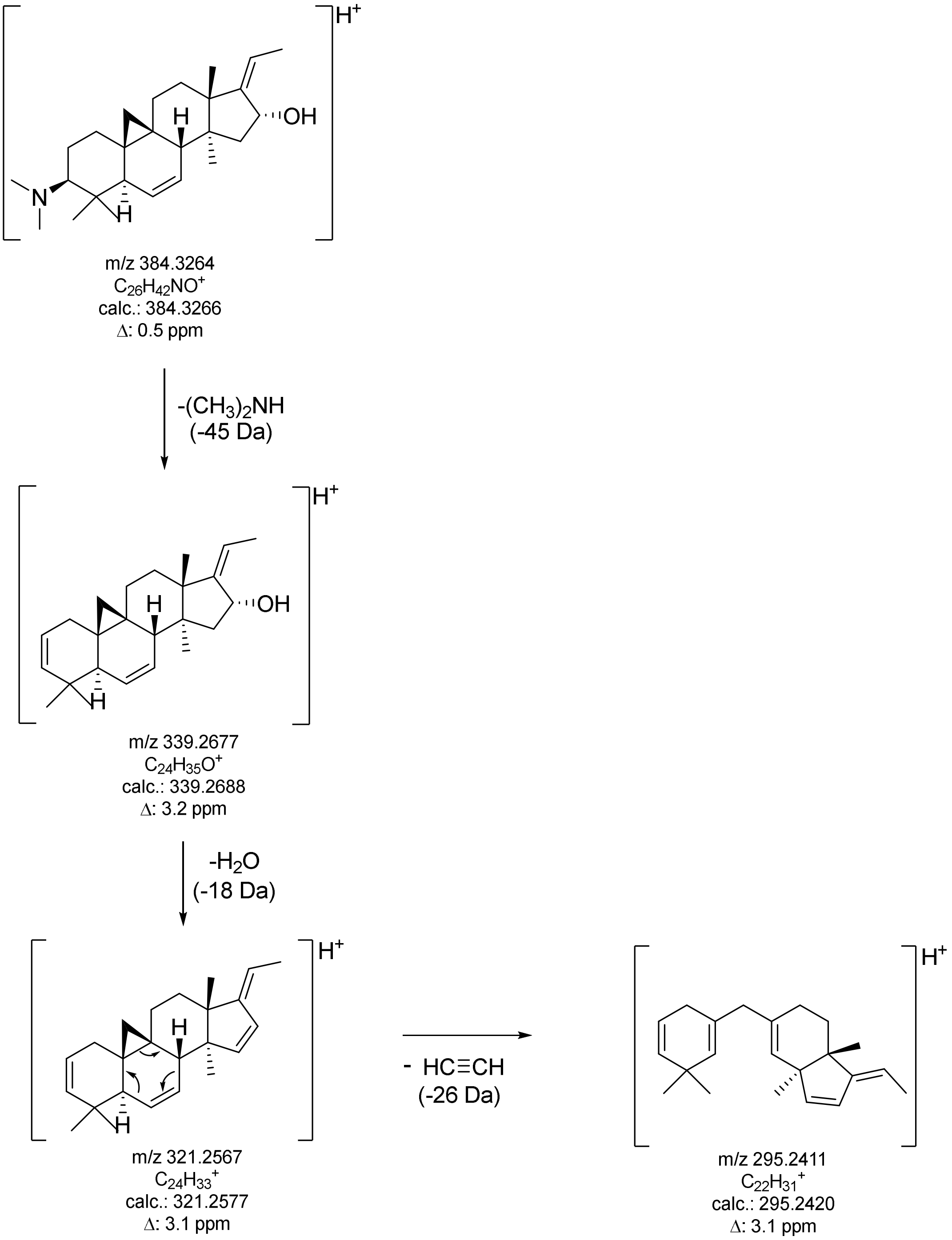

Figure S45. Possible fragmentation pathway of the $[M + H]^+$ ion of compound **36**. (The postulated position of the hydroxyl group is based on the already known structures of compound **12**).

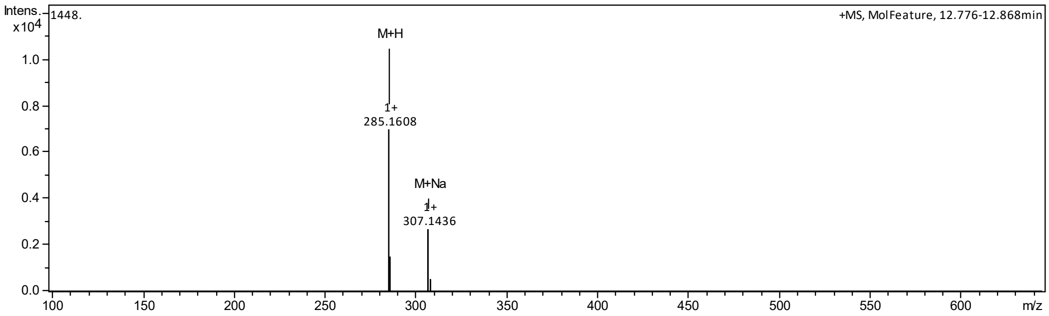

Figure S46. +ESI-QqTOF MS spectrum of compound **37**; m/z 285.1608 $[M+H]^+$ and 307.1436 $[M+Na]^+$.

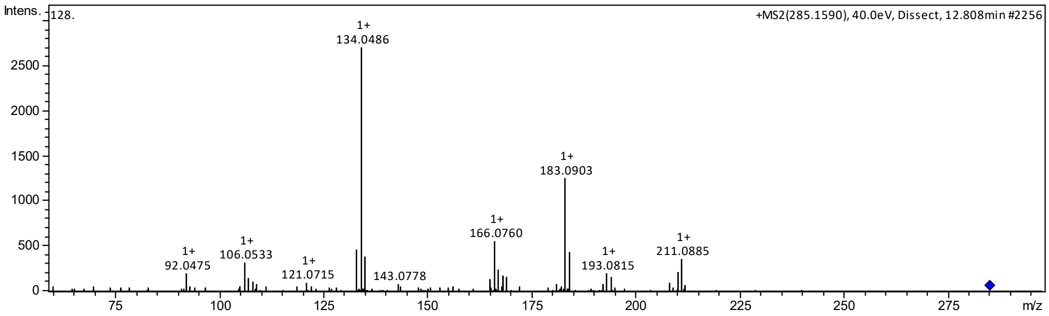

Figure S47. +ESI-QqTOF MS/MS spectrum of compound **37**.

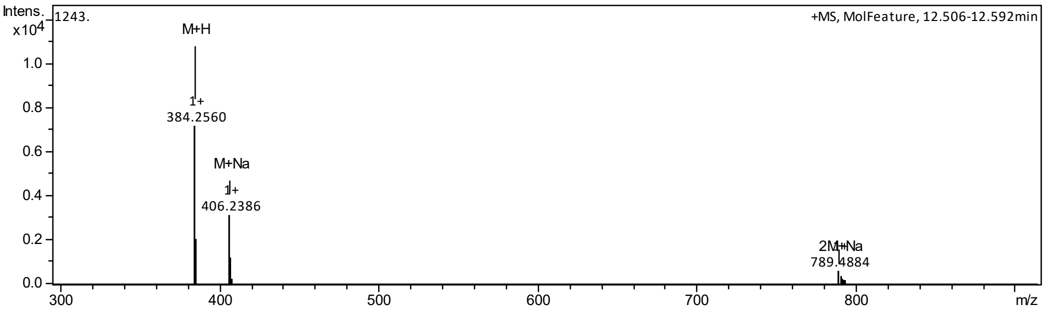

Figure S48. +ESI-QqTOF MS spectrum of compound **38**; m/z 384.2560 $[M+H]^+$, 406.2386 $[M+Na]^+$ and 789.4884 $[2M+Na]^+$.

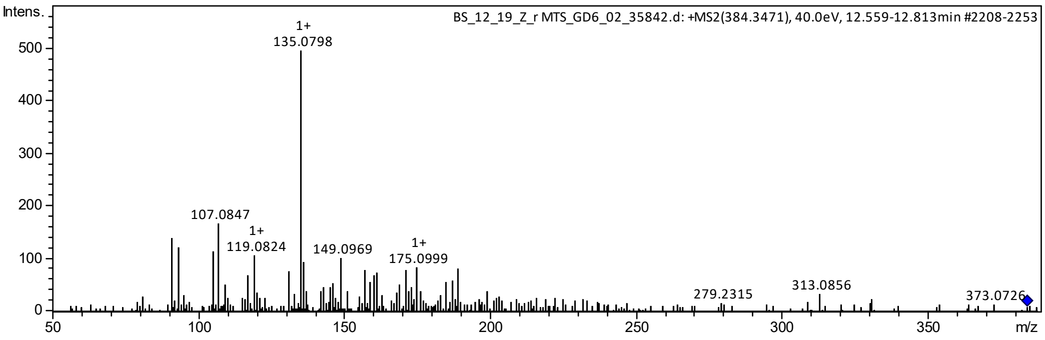

Figure S49. +ESI-QqTOF MS/MS spectrum of compound **38**.
